# Coronary artery bypass as a treatment of stent dislodgement: A case report

**DOI:** 10.1016/j.amsu.2019.09.013

**Published:** 2019-10-01

**Authors:** Khalid S. Ibrahim, Nizar R. Alwaqfi, Rashid K. Ibdah

**Affiliations:** a Division of Cardiac Surgery, Departmrent of General Surgery and Urology, College of Medicine, Jordan University of Science and Technology, Jordan; b Division of Cardiology, Departmrent of Internal Medicine, College of Medicine, Jordan University of Science and Technology, Jordan; c Princess Muna Center for Heart Diseases and Surgery, King Abdullah University Hospital, Jordan

**Keywords:** Stent, Dislodgement, CABG

## Abstract

**Introduction:**

Stent dislodgement is a known complication during coronary angiography. Different methods are used to retrieve it including open heart surgery.

**Case presentation:**

A 71 year-old male with stable angina was scheduled for elective coronary angiography. Angiography showed two significant stenosis: one in the proximal right coronary artery (RCA) and one in the left anterior descending artery (LAD). Upon deployment of the right coronary stent, it got lodged and the cardiologist was unable to retrieve it. The patient started to experience angina and his ECG showed ST segment elevation in the inferior leads. Emergency CABG was performed.

**Conclusion:**

Stent dislodgement is a rare but serious complication. Most cases are treated by interventional methods; however, CABG is still needed in some cases.

## Highlights

• Stent dislodgement is not a common complication of coronary angiogram.
• Dislodged stents are usually removed by catheterization.
• Coronary artery bypass surgery CABG is needed if retrieval is not successful.

## 1 Introduction

Iatrogenic injuries have accompanied percutaneous coronary intervention (PCI) since the beginning of this modality [1]. In the context of increasing number of complex percutaneous coronary interventions (PCI), dislodgement of an unexpanded coronary stent is a complication that, although rare, still happens [2]. The incidence of lost stent or other devices has dropped from 8.3% to 0.21% due to improvements in the equipment used [[3], [4], [5]]. However, when it happens, it is a serious complication that holds a risk of coronary or systemic thromboembolization, need for emergency coronary artery bypass surgery (CABG), or even death [2,3].

Different retrieval techniques of unexpanded stents have been implemented with a success rate as high as 86%. These techniques include use of balloon catheters (inflation within or distal to the lost stent), myocardial biopsy or biliary forceps, two twisted guide wires, basket devices, loop snares, etc. Nevertheless, surgical removal can still be required in a few cases [[4], [5], [6], [7]]. In this case, we reported a case that needed CABG as a treatment of stent dislodgement. This work has been reported in line with the SCARE criteria [8].

## 2 Case presentation

**Fig. 1:**
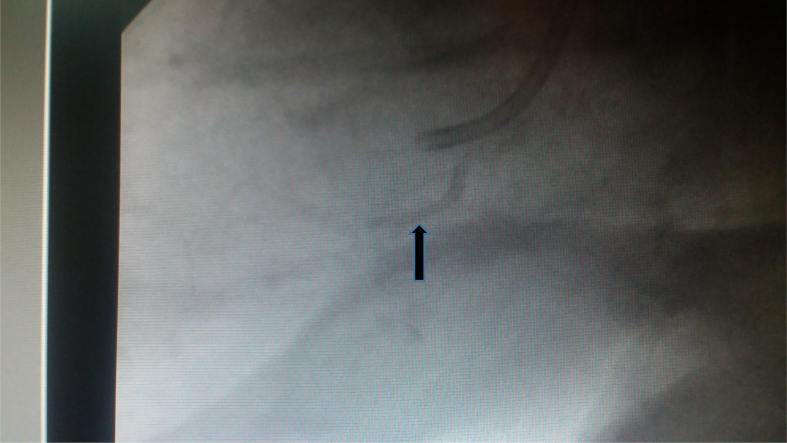
Shows the stent as lodged in the right coronary artery (Arrow) and the guidewire withdrawn.

**Fig. 2:**
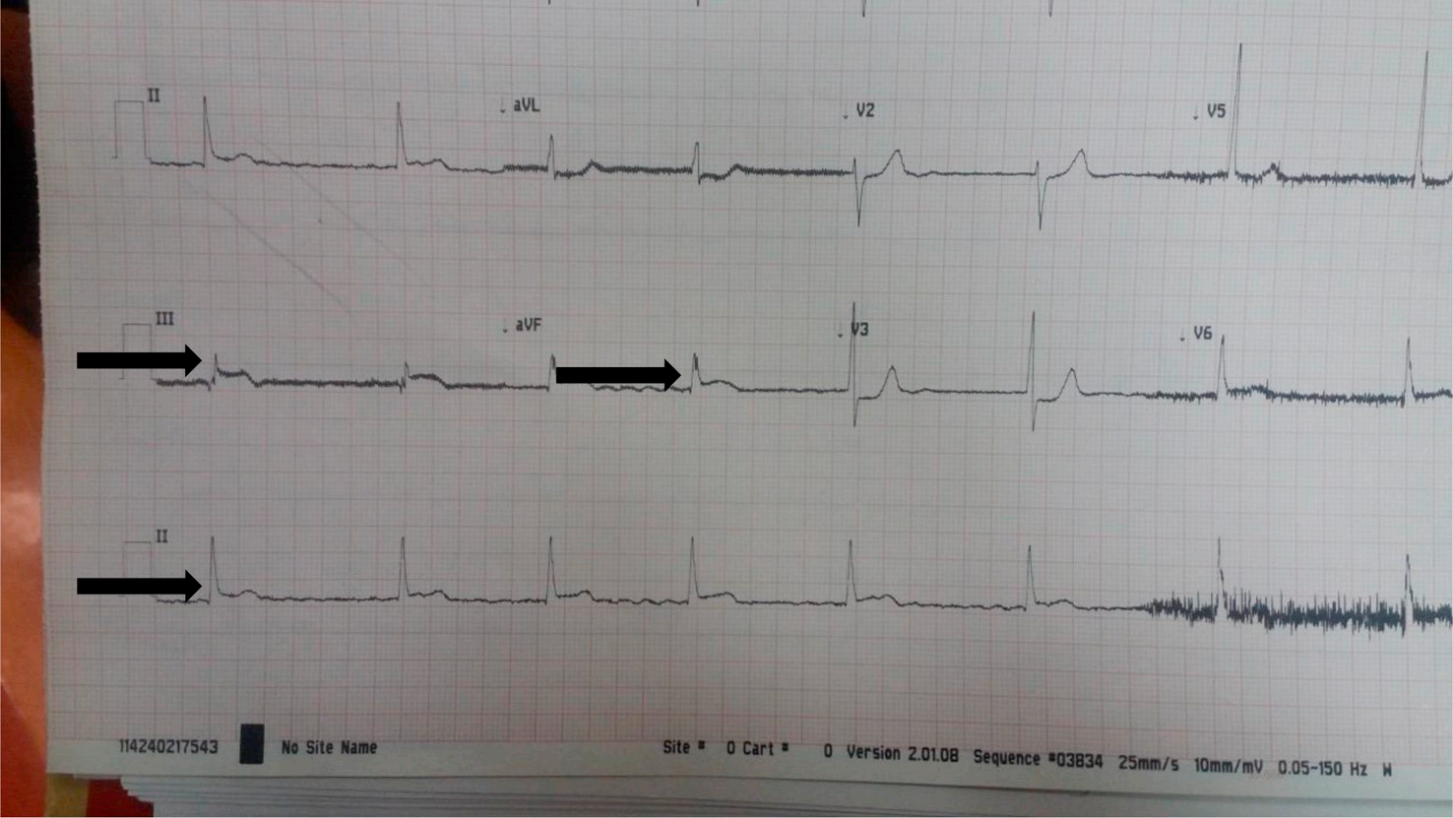
An Electrocardiogram of the patient showing ST segment elevation at inferior leads (Arrows).

In this report, we present a case of a 71 year-old male who was scheduled for elective coronary angiography for stable angina (Done by senior cardiologist). Angiography showed two significant stenosis; one in the proximal right coronary (75%) and one in the left anterior descending artery (75%). Upon deployment of the right coronary stent and before inflation, it lodged in the proximal part of the right coronary artery and the cardiologist was unable to retrieve it (Fig. 1). The guidewire was pulled accidentally and attempts to reinsert it failed. The patient started to have chest pain and his ECG showed ST segment elevation at inferior leads (Fig. 2). Cardiac surgery was consulted and emergency on pump CABG was performed with LIMA-LAD and SVG to RCA. The stent was not retrieved. He spent two nights in the cardiac intensive care unit and was discharged three days later. His postoperative course was uneventful. Thirty days follow up in the outpatient clinic was also uneventfull. Clopidogril (75mg daily) was given to the patient aiming at preventing embolization. The patient has given consent for possible publication of this case report.

## 3 Discussion

The complexity of PCI has increased and yet, the incidence of stent or other devices loss has significantly declined in recent years, probably due to improvement in equipment design, technology, and universal use of pre-mounted stents [2,5,6]. Stents were the most commonly lost devices and recent publications report stent loss incidence is as low as 0.21% [2,6]. In the literature, the most common cause of stent loss was attempts to deliver a stent through a previously deployed stent [6, 9] or when the stent-balloon assembly was pulled back into the guiding catheter proximal to the target lesion [4,5]. Poor support of the guiding catheter or guide wire, vessel tortuosity proximal to the lesion or severe vessel calcification and long stent may also predispose to stent entrapment 6).

A stripped stent may cause intracoronary or systemic embolization, thrombus formation, emergent coronary artery bypass graft surgery, or death [4,5]. The most commonly used technique was the small balloon technique, which is the simplest retrieval technique (5). This technique requires a retained guide wire in the slipped stent which allows advancement of the retrieval balloon into the entrapped stent [4,5].

**Fig. 3:**
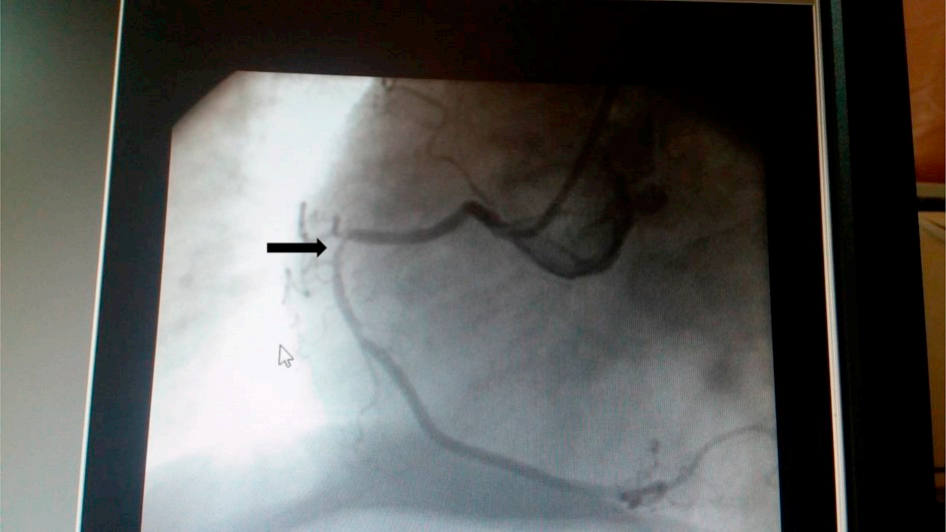
Right coronary angiogram showing acute angulation of the artery (Arrow).

In our case, the cause of the dislodgement was the acute angulation of the already atherosclerosed coronary artery according to the cardiologist (Fig. 3). First, the guidewire was inserted and balloon dilatation was performed. After that, the stent was inserted but missed proximal to the lesion. Unfortunately, the guidewire was accidentally pulled out. Another guide wire was inserted in an attempt to either insert a balloon to inflate it distal to the stent to pull it out or to crush the dislodged stent against the coronary wall with another stent. The new guide wire was unable to pass the dislodged stent and the patient became agitated and started to have chest pain and became hypotensive. The ECG showed ST segment elevation (Fig. 3). Patient was transferred to CCU and emergency CABG was performed the postoperative course was uneventful and the patient was discharged on postoperative day 5. Management of the patient and writing the case report was done in light of SCARE guidelines [8].

## 4 Conclusion

Stent dislodgement is a rare but serious complication. Most cases are treated by interventional methods; however, CABG is still needed in some cases. Presence of cardiac surgery backup in the same hospital is considered critical to the outcome of these patients. Clopidogrel was added to avoid thromboembolization from the existing stent.

## Ethical approval

Approved by the IRB committee at King Abdullah University Hospital.

## Sources of funding

No any source of funding.

## Author contribution

First author KI: study concept or design, writing the paper.

Co Author NW: Edition of the paper.

Co Auther RI: data collection, Edition of the paper.

## Trial registry number

1. Name of the registry: NONE
2. Unique Identifying number or registration ID: NONE
3. Hyperlink to the registration (must be publicly accessible): NONE

## Guarantor

Khalid Ibrahim.

Nizar Waqfi.

Rashed Ibdah.

## Consent

The patient has given consent for possible publication of this case report.

## Provenance and peer review

Not commissioned, externally peer reviewed.

## Declaration of competing interest

There is no conflict of interest for any author.

## Appendix A Supplementary data

SCARE Guideline Checklist SCARE Guideline Checklist
